# Exploring the Muco-Microbiotic Interface as a Hub for Microbial Metabolites and Immune Regulation in Gastroenteric Health and Disease

**DOI:** 10.3390/cells15010045

**Published:** 2025-12-25

**Authors:** Adelaide Carista, Melania Ionelia Gratie, Enrico Tornatore, Salvatore Accomando, Giovanni Tomasello, Domiziana Picone, Stefano Burgio, Francesco Cappello

**Affiliations:** 1 Department of Biomedicine, Neurosciences and Advanced Diagnostics (BiND), University of Palermo, 90127 Palermo, Italy; adelaide.carista@unipa.it (A.C.); melaniaionelia.gratie@unipa.it (M.I.G.); giovanni.tomasello@unipa.it (G.T.); domiziana.picone@unipa.it (D.P.); francesco.cappello@unipa.it (F.C.); 2 Department of Biological, Chemical and Pharmaceutical Sciences and Technologies (STEBICEF), University of Palermo, Viale delle Scienze Ed. 16, 90128 Palermo, Italy; enrico.tornatore@unipa.it; 3 Department of Health Promotion, Mother and Child Care, Internal Medicine and Medical Specialities “G. D’Alessandro”, University of Palermo, 90127 Palermo, Italy; salvatore.accomando@unipa.it; 4 Department of Medicine and Surgery, Kore University of Enna, 94100 Enna, Italy

**Keywords:** mucus layer, microbiota, mucin, host–microbe interactions, gastric mucosa, intestinal barrier, mucosal immunity, *H. pylori* infection, inflammatory bowel diseases, colorectal cancer

## Abstract

**Highlights:**

**What are the main findings?**

- The gastrointestinal mucus is a dynamic ecosystem where mucins, mucus-associated microbiota, and extracellular vesicles form an integrated functional unit, defined here as the muco-microbiotic (MuMi) layer.
- The MuMi layer shows strong regional specialisation along the gastroenteric tract, shaping site-specific microbial niches, metabolite production, and immune interactions.

**What are the implications of the main findings?**

- Viewing mucus, microbiota, and vesicular signalling as a single MuMi layer provides a unifying framework to better understand mucosal homeostasis and disease mechanisms, i.e., pathophysiology.
- This integrated perspective may improve experimental models and support the development of diagnostic and therapeutic strategies aimed at restoring mucosal barrier function in gastrointestinal disorders.

**Abstract:**

The mucus layer covering the gastrointestinal tract forms a specialised interface where mucins, microbes, and extracellular vesicles create a dynamic, self-regulating ecosystem. Here, we introduce the concept of the muco-microbiotic layer as an integrated eco-physiological system that maintains mucosal homeostasis through coordinated structural, metabolic, and immune functions. The MuMi layer varies regionally in its biochemical composition, microbial inhabitants, and environmental parameters—from the acidic stomach to the anaerobic colon—thereby generating distinct niches for microbial colonisation and metabolite production. We summarise current evidence on how mucin glycans, mucus-associated microbiota, and vesicle-mediated signalling sustain barrier integrity, nutrient flux, and immune tolerance. Perturbations in any of these components lead to barrier failure, microbial encroachment, and inflammation, contributing to a broad spectrum of disorders, including gastritis, inflammatory bowel disease, colorectal cancer, and metabolic syndrome. Methodological advances such as organoid and mucus-on-chip models, spatial multi-omics, and vesiculomics are now enabling site-specific analyses of this complex system. Conceptually, defining the mucus, microbiota, and vesicular compartments as a single MuMi layer provides a new framework for understanding mucosal physiology and pathophysiology, emphasising the interdependence between structure and function. Integrating this perspective into experimental and clinical research may open new avenues for diagnostics and therapies targeting mucosal health.

## 1. Introduction

The mucus layer that coats the epithelial surfaces of the gastroenteric tract constitutes a dynamic, gel-like extracellular matrix that separates resident microbial communities from host tissues while simultaneously serving as a nutrient niche, a signalling site, and a biophysical barrier. Here, the term “microbial communities” encompasses bacteria, viruses (including bacteriophages), and fungi; however, bacteria quantitatively and functionally dominate host–microbe interactions at the mucus–epithelial interface. For this reason, the present review focuses primarily on bacterial components populating in the mucus layer, while the roles of the mycobiome and virome are only briefly acknowledged and are more extensively addressed in dedicated literature. Moreover, throughout this review, the term “microbiota” refers to the assemblage of microorganisms inhabiting the mucus layer, whereas “microbiome” denotes their collective genetic and functional potential. The term “microbiotic,” as used in the title, reflects an integrated functional perspective as discussed in subsequent sections.

Although much of the literature has focused on the intestinal mucus, mucus–microbiota interfaces are found throughout the gastroenteric tract—from the acidic stomach to the small and large intestines—and display site-specific biochemical compositions, microbial assemblages, and functional properties [1]. Recognising these regional differences is critical when framing the mucus and its associated microbiota as a single, integrated functional unit, hereafter referred to as the muco-microbiotic (MuMi) layer of the gastroenteric tract [2]. This composite layer can be conceptually viewed as comprising three intimately interacting components: (1) the mucus matrix, formed mainly by secreted mucins and associated macromolecules; (2) the adherent and mucus-embedded microbiota, representing the biological interface with the host; and (3) a dynamic population of extracellular nanovesicles of both host and microbial origin, which mediate molecular exchange and cross-kingdom signalling within the MuMi layer [3,4].

As mucins represent the primary structural and biochemical scaffold of the mucus layer, their molecular diversity and regional specialisation provide an essential framework for understanding the organisation and function of the MuMi layer. At the molecular level, the principal gel-forming mucins differ between the stomach and the intestine: the gastric surface mucus is dominated by MUC5AC (with MUC6 in deeper gastric gland mucus), whereas the intestinal mucus is largely built on MUC2, with further regional variation between the small intestine and the colon [5]. These mucins confer distinct rheological and biochemical properties; for example, stomach mucus must withstand acidity and proteolysis, while colonic mucus forms a dense two-layer organisation (an almost bacteria-free inner layer and a colonised outer layer) that is essential for the spatial segregation of microbes from the epithelium. Thus, the molecular scaffold of the MuMi layer differs along the tract and helps shape local microbial niches.

Consistently, the composition and ecology of mucus-associated microbiota vary by site. The gastric MuMi layer—influenced strongly by pH, host secretions and common colonisers such as *Helicobacter pylori* [6] as well as other facultative taxa—shows a community structure distinct from that of the small intestine or colon, where obligate anaerobes (e.g., members of Bacteroides, Firmicutes) and specialist mucin degraders (e.g., *Akkermansia*, certain *Bacteroides* spp.) are more prevalent [7]. Regional differences in mucin glycans, mucus thickness, and turnover rate modulate substrate availability and, therefore, select for different functional guilds of microbes within the MuMi layer [8].

A defining feature of the MuMi layer is the local generation and action of microbially derived metabolites and extracellular nanovesicles within the mucus niche. Short-chain fatty acids (SCFAs), indole derivatives, secondary bile acids, and other small molecules are produced or concentrated at the mucus–epithelial interface, exerting potent effects on epithelial metabolism, barrier function, and mucosal immune responses. Among these, tryptophan-derived metabolites, including indole compounds and serotonin-related pathways, represent a paradigmatic example of host–microbe co-metabolism at the MuMi interface. While gut-derived serotonin is classically produced by enteroendocrine cells and modulates epithelial and immune functions, emerging evidence indicates that specific gut bacteria can also contribute to serotonin and serotonin-like metabolite pools, thereby influencing both mucosal immunity and microbial community structure [9]. Moreover, bacteria-derived extracellular vesicles (EVs) and host EVs within mucus can carry proteins, nucleic acids, and signalling lipids that mediate interkingdom communication; importantly, the cargo and abundance of these nanovesicles are likely to vary between gastric and intestinal MuMi layers because of differences in microbial community composition and luminal environment [10].

Perturbations of the MuMi layer—whether via altered mucin expression/glycosylation, dysregulated mucin-foraging by microbes, shifts in mucus-associated metabolites, or changed EV dynamics—have been associated with diverse gastroenteric pathologies. Gastric MuMi alterations (for example, those linked to *Helicobacter pylori* (*H. pylori*) colonisation and gastric carcinogenesis or those related to or *Giardia Lamblia* (*G. Lamblia*) infection in the duodenal region, determining intestinal and extraintestinal manifestations, such as, respectively, diarrhoea and asthenia) differ mechanistically and ecologically from colonic MuMi disturbances implicated in inflammatory bowel disease or associated barrier dysfunction conditions [4,11,12]. Therefore, while the MuMi layer provides a unifying conceptual framework, mechanistic and translational studies must respect regional specificity and rely on site-appropriate models and sampling strategies.

Methodological advances now allow more precise interrogation of MuMi layers across the gastroenteric tract [13]: region-targeted mucus sampling, high-resolution imaging, glycomics and spatial metabolomics, refined in vitro systems (organoids and organ-on-chip platforms) and EV-profiling techniques can reveal local metabolite landscapes, microbial spatial organisation and host–microbe signalling events that bulk luminal assays miss. Nonetheless, standardisation of sampling protocols, better in situ quantification of metabolites and EVs, and comparative studies that explicitly contrast gastric versus intestinal MuMi layers remain pressing needs.

In this review we adopt the term MuMi layer of the gastroenteric tract and critically synthesise current knowledge on (1) the structural and biochemical heterogeneity of mucus along the tract; (2) site-specific colonisation strategies and mucin foraging by microbes; (3) the repertoire and local actions of mucus-associated metabolites and nanovesicles; and (4) implications for regional immune regulation and disease. Where high-quality, site-specific data exist [14], we differentiate the gastric MuMi layer from its intestinal counterparts; where data are lacking, we highlight the gap and propose experimental approaches to resolve it. Our goal is to provide a comparative, translationally oriented framework that can guide future investigations and therapeutic strategies targeting MuMi layers in distinct gastroenteric niches.

The MuMi layer may also be considered a primary actor of innate immunity, preserving host integrity and protecting the inner compartments from external threats. Its composition varies along the gastrointestinal tract to meet distinct biological needs and local functional demands. Beyond barrier functions, the MuMi layer interacts with the adaptive immune system, facilitating crosstalk with the resident microbiota. The selective colonisation of the stomach by microbes such as *H. pylori* or the duodenum by *G. lamblia*, as mentioned, likely reflects their ability to overcome regional MuMi layer defences, shaped by factors such as gastric acid, bile, and pancreatic secretions. In the sections that follow, we expand on these concepts and their implications for gastrointestinal physiology and disease.

## 2. The Gastroenteric Mucus Barrier: Regional Structure and Composition

The gastroenteric mucus barrier is not a homogeneous entity along the alimentary canal, but rather a regionally differentiated system whose structural, biochemical and rheological properties adapt to the specific micro-environment of each segment. In the stomach, the secreted gel layer must withstand strong acidity (pH ≈ 1.5–3.5), pepsin activity and mechanical shear, and is therefore composed predominantly of gel-forming mucins such as MUC5AC and MUC6, secreted by surface mucous cells and gland neck cells [15,16]. Beneath this layer lies an unstirred bicarbonate-rich fluid that helps maintain a near-neutral pH at the epithelial surface, thereby creating a micro-climate of protection [17].

In contrast, the small and large intestine display distinct mucus architectures. The small intestine typically presents a single, loosely adherent mucus layer, facilitating nutrient absorption while offering moderate microbial exclusion. By contrast, the large intestine exhibits a classic two-layer (or multilayer) organisation: an inner, firmly adherent mucus layer (mainly composed of MUC2) that is nearly devoid of bacteria, and an outer, more permeable layer that is colonised by microbial communities [18,19]. The thickness, turnover rate, and glycosylation pattern of the mucins differ markedly between these regions, reflecting adaptation to factors such as luminal flow dynamics, microbial density, substrate availability, and host secretions [7,20].

At the molecular level, the mucin glycan repertoire influences the gel’s physical properties and the substrate landscape available to mucus-associated microbes. Highly glycosylated “bottle-brush” mucins carry varied glycan moieties (sialylated, sulfated, fucosylated, etc.) whose relative abundance shifts along the tract, e.g., a greater degree of sulfation in colonic mucins compared to small-intestinal ones [14,21]. These biochemical differences result in distinct viscoelastic properties: the gastric mucus gel, formulated to resist acid hydrolysis and proteolysis, displays a more compact and cross-linked matrix, whereas intestinal mucus tends to be more hydrated and dynamic, supporting both lubrication and microbial ecosystems.

Moreover, the microenvironmental conditions, such as pH, ionic strength, mucin concentration, and shear stress, vary substantially along the gastroenteric tract. For instance, the pH gradient from lumen to epithelium in the stomach requires a gel that limits proton diffusion, whereas in the colon microbial metabolism generates local changes in pH, redox potential, and metabolite concentration, which further modulate mucus structure and turnover [13]. Consequently, regional heterogeneity in the mucus barrier creates distinct ecological niches for the mucus-embedded microbiota, influences metabolite gradients, and shapes host–microbe interactions in site-specific ways.

Together, these observations underscore that when considering the MuMi layer of the gastroenteric tract, it is essential to recognise the regional specialisation of the mucus component—its architecture, mucin composition, rheology and dynamic renewal—as the foundational scaffold upon which microbial and vesicular components build their functional niche.

**Figure 1:**
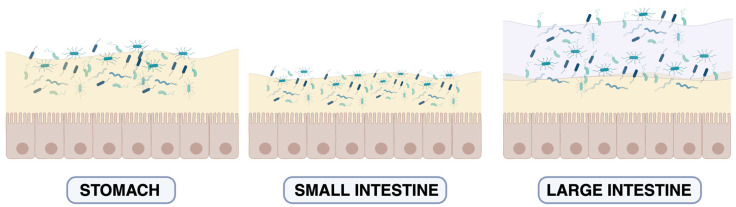
Schematic view of the MuMi layer in the gastroenteric tract. Schematic representation of the mucus layer and microbial density in the stomach, small intestine, and large intestine. In the stomach, a single mucus layer provides protection from gastric acid and supports limited microbial colonisation. In the small intestine, the mucus layer is thinner and rapidly renewed, allowing occasional and controlled microbial contact with the epithelial surface. In the large intestine, a two-layered mucus structure is observed: an inner, dense, largely sterile layer (light yellow) and an outer, loose layer (purple) that harbours a dense, diverse microbial community. This organisation reflects regional specialisation in barrier function and host–microbe interactions along the gut. For more information: [18,22]. The colour variation in bacterial cells across the panels reflects regional differences in microbial diversity: transient and heterogeneous populations in the stomach and small intestine versus a dense, compositionally stable community in the large intestine. For more information: [23,24].

To better understand this new paradigm, Figure 1 schematically illustrates the MuMi layer across the various tracts of the gastrointestinal tube. Table 1 and Table 2 summarise the main structural and functional characteristics of the MuMi layers in these different tracts of the alimentary canal.

**Table 1:** Structural Characteristics of the Gastroenteric MuMi Layer. Comparison of main structural features of the MuMi layer along the gastroenteric tract, including predominant mucins, layer architecture, and microbiota composition with environmental context.

Gastroenteric Region	Main Mucins/Mucus Composition	Mucus Architecture ^1^	Microbiota Features	Environmental Features	References
**Stomach**	MUC5AC (surface epithelium), MUC6 (glandular)	Single thick adherent gel layer; pH 1.5–3.5	Low microbial density; acid-tolerant taxa such as *H. pylori* and facultative bacteria	Extreme acidic environment; physical barrier for epithelial protection	[15,25,26]
**Small Intestine**	Predominantly MUC2	Single loosely adherent layer; thinner than large intestine	Moderate microbial density; mixture of anaerobes and facultative bacteria; partial mucin degradation	Nutrient absorption; moderate microbial exclusion; dynamic environment	[1,5]
**Large Intestine**	Predominantly MUC2	Two layers: inner dense adherent layer (almost bacteria-free), outer colonised layer	High microbial density; obligate anaerobes	Supports dense microbiota; strong barrier function; site-specific metabolic activity	[18]

^1^ “Mucus architecture” refers to layer number, adhesion strength, thickness, and relative stratification.

**Table 2:** Functional Features of the Gastroenteric MuMi Layer. The functional characteristics of the MuMi layer, including turnover rates, glycosylation patterns, metabolite support, and interaction with local immune cells, highlight regional differences along the gastrointestinal tract.

Gastroenteric Region	Functional Reference	Estimated Turnover	Predominant Glycosylation/Glycans	Local Immune Interactions	References
**Stomach**	Acid and protease resistance; separation of microbes from epithelium	Hours to ~1 day	Surface MUC5AC is predominantly sialylated, whereas glandular MUC6 is enriched in sulfated glycans	Local innate immunity; TLR-mediated recognition of *H. pylori*	[17,25]
**Small Intestine**	Nutrient absorption; microbial modulation	4–6 h	Mixed O-glycans, moderate sulfation	Interactions with Peyer’s patches; mucosal immune sampling	[1,5]
**Large Intestine**	Barrier function; supports microbial metabolism; metabolite gradients	1–2 days	High sulfation and fucosylation; complex glycans on MUC2	Dense immune surveillance; modulation of Tregs and IgA production	[18]

## 3. The Mucus-Associated Microbiota: Diversity and Functional Niches

Within the gastroenteric MuMi layer, the microbiota located in the mucus milieu—or “mucus-associated microbiota (MAM)”—constitutes a distinct ecological community near the epithelial surface. Unlike the luminal microbiota captured in faeces, MAM is embedded within the mucus gel, has unique adhesion and metabolic capabilities, and exhibits both compositional and functional divergence from the broader luminal microbiota. For example, spatial analyses show that the mucus-embedded community forms a dense band over the colonic mucosa, excluding many Proteobacteria taxa and enriching for anaerobic Clostridia species, indicating that it is stable and specialised [27]. This distinctive community thereby occupies a niche in which substrate availability (mucin glycans), host secretions, and metabolite gradients foster a microbiota adapted for close host interactions.

The diversity of the mucus-associated microbiota reflects adaptation to glycan-rich, low-oxygen microenvironments and is shaped by mucin composition, mucus structure and regional microenvironmental parameters. Many mucin-degrading bacteria harbour specialised glycoside hydrolases (GHs). For instance, a recent systematic study of human gut microbes found that numerous genera across the *Verrucomicrobia*, *Bacteroidetes,* and *Firmicutes* phyla encode multiple mucin-degrading GHs [28,29]. Such enzymatic repertoires enable microbes like *Akkermansia muciniphila*, *Bacteroides thetaiotaomicron* and members of *Ruminococcus* torques to forage on host-derived mucin glycans, creating niche-specific microbial guilds that differ from those in the gut lumen [30].

Regional variation along the gastroenteric tract further influences MAM composition and functional traits. The stomach, under low pH and high proteolytic stress, is colonised by acid-tolerant and facultative taxa, whereas the small intestine and colon provide environments richer in mucin glycans, slower transit and higher microbial density. Empirical studies show that the mucus-associated layer in the colon is more diverse and includes species not found in lumen samples [19,27]. Moreover, mucin glycan patterns—including fucosylation, sialylation and sulfation—differ between gut regions and thereby select for microbes with corresponding glycosyl hydrolase repertoires. For example, A. muciniphila’s sialidases and fucosidases are essential for colonisation of mucin-rich layers [31]. Representative mucus-associated microbes and their functional roles are summarised in Table 3. The main regional differences in the mucus-associated microbiota along the gastroenteric tract are summarised in Table 4.

Functional niches within the mucus layer extend beyond simple nutrient access and include biofilm-like organisation, cross-feeding networks, and metabolite production. The mucus-associated microbiota forms cohesive communities that can enhance colonisation resistance and buffer against perturbations; e.g., the mucus-embedded community lost fewer *Clostridia* and better resisted *Proteobacteria* blooms during antibiotic treatment than luminal communities [27]. In turn, mucin-foraging microbes release monosaccharides and other metabolites that feed secondary degraders or pathogens, such as when mucin degradation by specialist taxa supports colonisation by *Clostridioides difficile* via liberated sugars [30]. Such trophic networks demonstrate that MAM is not a passive resident population but an active functional niche with implications for host–microbe symbiosis and dysbiosis.

Finally, the mucus-associated microbiota interacts intimately with host immune and barrier functions. Because MAM resides in immediate proximity to the epithelium, it can influence goblet cell function, mucus secretion, and immune signalling. For instance, alteration of the mucus layer in goblet-cell-specific knockout mice led to higher mucosal microbiota diversity, increased pathobiont abundance, and induction of inflammatory cytokines [32]. Given that MAM signatures differ from luminal profiles and correlate with diseases such as inflammatory bowel disease (IBD), irritable bowel syndrome (IBS) and colorectal cancer (CRC), the functional niche of MAM within the MuMi layer warrants translational attention.

Consideration of the mucus-associated microbiota invites a shift in microbial ecology modelling: rather than viewing the gut microbiota as a bulk lumen population, we must appreciate microenvironment-specific niches, such as MAM, and their sustained interactions with host physiological and pathological states. Future research should map MAM taxonomic and functional profiles longitudinally, compare mucus vs. lumen compartments across disease states, and integrate mucus sampling in microbiome studies to uncover niche-specific therapeutic opportunities.

**Table 3:** Representative Mucin-Associated Microbes and Their Functional Roles in the MuMi Layer. Key examples of microbes specialised for the mucus niche of the MuMi layer, their phylum classification, functional roles related to mucin utilisation and host interactions, and supporting evidence for their mucus association.

Microorganism	Phylum	Key Functional Attributes	Evidence for Mucus Association	Microorganism	References
***Akkermansia*** ***muciniphila***	*Verrucomicrobia*	Mucin specialist; produces SCFAs; stimulates epithelial renewal	Glycoside hydrolases specificity for sialic/fucose caps, growth on mucin media.	*Verrucomicrobia*	[31,33]
***Bacteroides thetaiotaomicron***	*Bacteroidetes*	Broad glycan-forager; supports cross-feeding networks	Recognised mucosa-associated glycan degrader.	*Bacteroidetes*	[34]
***Ruminococcus torques***	*Firmicutes*	Mucin degrader; implicated in IBD/CRC	Detected enriched in mucus layer in disease.	*Firmicutes*	[19]
***Clostridia*** **(various species)**	*Firmicutes*	Anaerobic niche stabilisers; abundant in mucus biofilm-like communities	Mucus-embedded communities enriched for Clostridia.	*Clostridia* (various species)	[27]

**Table 4:** Regional distribution and dominant taxa of the mucus-associated microbiota in the MuMi layer along the gastroenteric tract. The table summarises the main environmental parameters, the dominant microbial taxa, and the key functional traits characterising the mucus-associated microbiota across different segments of the gastroenteric tract. While the gastric environment supports a sparse and acid-tolerant community, the small intestine harbours facultative anaerobes adapted to bile and rapid flow, and the colon sustains a dense, anaerobic ecosystem enriched in mucin-degrading specialists.

Region	Environmental Features	Dominant Taxa (Examples)	Functional or Structural Features	References
**Stomach**	Acidic pH (1–3); oxygenated niche; MUC5AC-rich mucus	*H. pylori*, *Mycobacterium Tuberculosis*, *Streptococcus*, *Rothia*, *Veillonella*, *Lactobacillus* spp.	Limited colonisation; acid resistance; mucin adhesion; local modulation of mucosal immunity	[35]
**Small intestine**	pH 5–7; bile salts; high flow rate; lower mucin density	*Lactobacillus*, *Streptococcus*, *Enterococcus*, *Veillonella*, *Bacteroides* spp.	Facultative anaerobes; nutrient scavenging; transient colonisation	[36]
**Large intestine**	Neutral pH (~7); anaerobic; high mucin content (MUC2); slow transit	*A. muciniphila*, *B. thetaiotaomicron*, *Ruminococcus gnavus*, *Eubacterium rectale*, *Faecalibacterium prausnitzii*	Dense mucus-associated microbiota; expression of glycoside hydrolases and sialidases; high metabolic activity	[37]

## 4. The Novel Concept of the Muco-Microbiotic Layer

The traditional view of the mucus barrier as a passive physical coating separating microbes from host tissues is being progressively replaced by a more dynamic model. In this framework, the mucus, its associated microbiota, and nanoscale vesicular components form an integrated functional entity—the MuMi layer—that mediates continuous communication between the host epithelium and the luminal environment [3,4]. This paradigm shift builds upon recent insights into the biochemical complexity of mucins, the ecological adaptability of mucus-resident microbes, and the emerging role of immunological aspects of intestinal mucus and mucins [38].

Within the MuMi layer, bidirectional interactions occur across molecular, metabolic, and immunological levels. Mucins not only act as physical scaffolds but also provide selective nutrients and adhesion sites for commensals, while microbial metabolites and enzymes remodel the mucus matrix, modulating its viscosity and turnover [33]. In parallel, epithelial cells sense microbial products diffusing through the mucus and respond by adjusting mucin secretion, releasing antimicrobial peptides, and modulating immune tolerance [39]. A crucial component of this system is the population of EVs derived from both host and microbial cells. Bacterial EVs can carry enzymes, signalling molecules, and microbe-associated molecular patterns that influence epithelial gene expression and barrier function, while host-derived EVs convey immunoregulatory cues and antimicrobial factors back toward the luminal microbiota [40]. Through this vesicular traffic, the MuMi layer acts as a biochemical “interface” rather than a static frontier.

Functionally, the MuMi layer operates as an integrated eco-physiological system that maintains mucosal homeostasis. Its integrity ensures selective permeability, controlled microbial colonisation, and fine-tuned immune dialogue. Perturbations, such as dysbiosis, mucin depletion, or altered EV signalling, can propagate across the system, leading to inflammation, metabolic imbalance, or increased pathogen susceptibility [41,42].

Conceptually, defining the mucus, microbiota, and vesicular compartments as a single MuMi layer underscores the interdependence of structural and biological processes at the mucosal surface. This holistic model provides a novel framework for interpreting mucosal physiology and pathophysiology—bridging microbiology, glycobiology, and immunology into a unified mucosal systems biology. Future research should aim to delineate MuMi-layer dynamics in different gut regions and under varying physiological and disease contexts, using advanced imaging, -omics, and organ-on-chip approaches.

## 5. Functional Roles of the MuMi Layer

The MuMi layer of the gastroenteric tract serves as more than a passive coating: it acts as a multifaceted eco-physiological interface in which structural, metabolic and immunological processes cooperate to maintain mucosal homeostasis. By integrating the gel matrix of mucins, the microbial communities embedded within, and the vesicular and soluble signalling milieu, the MuMi layer orchestrates selective permeability, microbial containment and host–microbe communication at the mucosal surface. For instance, recent reviews highlight how mucin-glycan scaffolds and mucus dynamics shape microbial ecology, while microbial metabolites and host responses feedback on mucus biology [19]. As a morpho-physiological barrier, the MuMi layer limits direct contact between luminal microorganisms and epithelial cells while permitting the diffusion of nutrients, ions and signalling molecules. The mucin network acts as a size- and charge-selective sieve whose properties are modulated by local pH, ionic milieu and glycosylation state. This barrier function is essential not only for excluding pathogens but also for allowing colonisation by mucus-adapted commensals. When the barrier component is compromised—e.g., by mucin deficiency or increased mucin-degrading activity—the result is increased epithelial exposure to microbes and their products, setting the stage for inflammation [43].

Beyond its barrier role, the MuMi layer functions as a nutritional and signalling interface. Mucins and associated glycans provide selective substrates for mucus-resident microbes, such as *A. muciniphila* and *B. thetaiotaomicron*, whose glycoside hydrolase activities liberate monosaccharides, oligosaccharides, and fermentation products, including SCFAs. Notably, SCFAs also exert epigenetic effects, for example, through histone deacetylase inhibition, thereby promoting regulatory T cell differentiation and contributing to immune tolerance at the MuMi interface [44]. These microbial metabolites support epithelial energy metabolism, reinforce tight junction integrity, and influence host gene expression [33].

From an immunological standpoint, the MuMi layer constitutes the frontline of mucosal immune dialogue. Microbial antigens, metabolites and EVs can traverse the mucus matrix and epithelial barrier to engage epithelial and immune cells. Goblet-cell mucin secretion, antimicrobial peptide production and IgA transcytosis are influenced by signals emanating from mucus-associated microbes: for example, altered mucus integrity leads to changes in dendritic cell activation and shifts in T-cell differentiation, and this intimate proximity and signalling capacity enable modulation of immune tolerance and readiness simultaneously [45].

Critically, the MuMi layer is a dynamic, adaptive system. External factors, including dietary fibre intake, bile acid fluxes, antibiotic exposure, stress hormones, and luminal flow changes, affect mucus composition and turnover, microbial community structure, and vesicular signalling. For example, fibre-deprived diets lead to increased mucin-foraging microbes, thinning of the mucus barrier and enhanced pathogen access [46]. When adaptation fails or is overwhelmed, the system shifts from homeostasis to dysfunction: barrier breakdown, microbial translocation, immune activation and disease onset [47].

Together, these functional roles position the MuMi layer as a central integrative hub: not just a static barrier, but an active ecosystem interface that balances protection with mutualistic microbial habitation and adapts to internal and external perturbations. Understanding its mechanisms offers new insights into mucosal physiology and opens translational avenues for diagnostics, therapeutics and microbiome-targeted interventions.

## 6. Pathophysiological Implications

Disruption of the MuMi layer—whether by direct damage to the mucus scaffold, shifts in mucus-associated microbial communities, or altered vesicular signalling—has consequences that propagate across the epithelial–immune–microbial axis and manifest in a spectrum of diseases. Because the MuMi layer integrates mechanical protection, selective nutrient provisioning, local metabolite gradients, and signalling (including EV-mediated traffic), perturbations produce system-level effects, including barrier failure, microbial translocation, altered immune tone, and metabolic dysregulation. Below, we summarise the major disease domains in which MuMi-layer dysfunction may be implicated, and indicate putative mechanistic links supported by experimental and clinical studies.

### 6.1. Gastric Disease: H. pylori, Chronic Gastritis and Gastric Carcinogenesis

The gastric MuMi niche is a selective habitat shaped by extreme acidity, distinct mucins (MUC5AC, MUC6) and a low-density, specialised microbiota. *H. pylori* colonises the surface mucous gel and manipulates mucin expression (for example, downregulating MUC5AC), enabling closer epithelial access, chronic inflammation and progression along the gastritis–atrophy–metaplasia–cancer cascade [4,48]. Experimental and clinical studies demonstrate that *H. pylori* interacts with and modifies the gastric mucus, facilitating persistent colonisation and mucosal injury [49]. Moreover, altered mucin expression and mucin-related glycan landscapes are observed in premalignant and malignant gastric tissues [49], suggesting a potential link between MuMi layer changes and carcinogenesis.

### 6.2. Inflammatory Bowel Disease: Mucus Erosion, MUC2 Defects and Bacterial Encroachment

In ulcerative colitis and, to variable degrees, Crohn’s disease, defects in the colonic mucus barrier are among the most consistent mucosal abnormalities. Studies show thinning or patchy loss of the inner adherent mucus, abnormal MUC2 production/glycosylation, and increased bacterial penetration to the epithelial surface, changes that correlate with inflammation. In particular, landmark reviews and experimental data emphasise that mucus barrier defects increase epithelial exposure to microbes and microbial products, promoting aberrant immune activation in genetically predisposed hosts [50,51]. Interestingly, the experimental models proposed in these studies link specific microbiota configurations and dietary factors to mucus loss and colitis-like phenotypes.

### 6.3. Colorectal Cancer: Mucin Alterations, Microbial Niches and Tumour Microenvironment

Aberrant mucin expression, changes in glycosylation and increased mucinous differentiation are frequently observed not only in colorectal cancer but also in pre-neoplastic lesions. Particularly, the microbe–mucus interface is implicated in tumorigenesis through several mechanisms: (i) altered glycocode exposes or creates niches that select for pro-inflammatory or genotoxic microbes; (ii) mucin degradation products and microbial metabolites (e.g., secondary bile acids) can promote epithelial proliferation or DNA damage; and (iii) chronic mucus disruption fosters persistent inflammation, as summarised by recent reviews [52,53] focusing on how shifts in mucus composition and mucus-associated microbiota may contribute to colorectal cancer pathophysiology and influence therapeutic responses.

### 6.4. Metabolic Disease, Obesity and Systemic Effects

Emerging evidence suggests that MuMi layer integrity is linked to host metabolic phenotypes. Obesity-associated microbiota can contribute to defects in the colonic mucus barrier and related mucosal dysfunction, which in turn may exacerbate low-grade inflammation and metabolic impairment. Animal studies [54,55] demonstrate that obesity-prone microbial communities are associated with altered mucus properties; clinical associations link dysbiotic profiles with obesity and non-alcoholic fatty liver disease, suggesting a cross-talk between mucosal ecology and systemic metabolism. Mechanistically, the results of these studies suggest how changes in mucus-resident microbiota and their metabolites (e.g., altered SCFA profiles, secondary bile acid patterns) can modulate epithelial signalling, barrier function and systemic metabolic regulation.

### 6.5. Increased Susceptibility to Infection and the Role of Diet and Antibiotics

The MuMi layer is a frontline determinant of colonisation resistance. Dietary fibre deprivation experiments powerfully demonstrate that when dietary microbiota-accessible carbohydrates decrease, mucin-degrading bacteria proliferate and erode the colonic mucus, increasing vulnerability to enteric pathogens [43]. Similarly, antibiotic exposures can profoundly perturb mucus structure, leading to mucus erosion and pathogen overgrowth in experimental models [56]. Even bacterial taxa traditionally regarded as commensals—such as coagulase-negative staphylococci—may display multidrug-resistant phenotypes and harbour biofilm-associated genes, as shown in a recent study on isolates from ovine milk [57]. These traits can enhance their persistence and competitive fitness within mucosal niches, particularly when the MuMi-layer barrier is compromised.

These results show that relatively common environmental factors can rapidly compromise MuMi integrity, creating windows of pathogen susceptibility.

### 6.6. Ageing, Mucus Thinning and Immune Consequences

Ageing is associated with quantitative and qualitative changes in the MuMi layer: several studies in mice and humans report reduced colonic mucus thickness, altered goblet cell function and shifts in mucus glycosylation with age, accompanied by increased epithelial contact with bacteria and low-grade inflammation. These alterations may contribute to the phenomenon of inflammaging and to age-associated susceptibility to infection and metabolic decline; conversely, certain mucus-colonising commensals (e.g., *A. muciniphila*) have been shown experimentally to restore mucus thickness and ameliorate age-related mucosal changes [58,59].

### 6.7. Extracellular Vesicles: Mediators and Biomarkers in Disease

EVs—from host cells, commensals or pathobionts—are increasingly recognised as important mediators of MuMi-layer signalling in health and disease. Microbial EVs carry enzymes, toxins, and signalling molecules that can modulate mucin structure, epithelial gene expression, and immune responses; host EVs carry antimicrobial peptides, signalling RNAs, and immune modulators that shape microbial communities [60,61]. Emerging evidence indicates that microbial EVs can modulate mucosal barrier properties, for example, by influencing mucin glycosylation and mucus viscoelasticity, thereby reshaping the physical architecture of the MuMi layer and its ability to exclude opportunistic pathogens [62]. Both host- and microbe-derived EVs are also expected to contribute to colonisation resistance by influencing microbial competition dynamics and promoting niche stability within the mucus-associated community, consistent with the central role of mucus and mucin-type O-glycans in guiding host–microbe symbiosis [63]. Since alterations in EV cargo and flux have been associated with gastrointestinal diseases, EVs are being explored as both biomarkers and therapeutic vehicles to restore mucosal homeostasis.

### 6.8. Translational Implications: Diagnostics, Prevention and Therapy

Recognition of the MuMi layer as a pathophysiologically central entity suggests multiple translational angles: (i) biomarkers—mucus-associated microbial signatures, mucin glycoforms or EV cargo could become diagnostic/prognostic markers for IBD, CRC or metabolic risk; (ii) preventive strategies—dietary interventions (e.g., adequate fibre) that preserve mucus integrity; (iii) microbiome-directed therapies—targeted probiotics (e.g., *A. muciniphila*), prebiotics, or ecological therapeutics aimed at restoring mucus-friendly communities; (iv) EV-based interventions—engineered vesicles delivering mucin-stabilising factors or anti-inflammatory cargo. Early preclinical data support some of these approaches; however, human trials remain limited, and the mechanistic understanding is incomplete [43,54].

Key gaps remain: how do specific mucin glycoforms tune microbial community assembly at high resolution? What are the kinetics and molecular determinants of EV trafficking within the MuMi layer? Can we develop minimally invasive assays to sample mucus-resident microbiota and EVs in humans reliably? Longitudinal, region-specific sampling, high-resolution imaging, single-cell and single-vesicle ‘omics, and organ-on-a-chip models will be essential to move from correlative studies to causal mechanistic insights. Indeed, at the best of our knowledge, no approved drugs selectively target specific mucin classes (e.g., MUC2 or MUC5AC). Nevertheless, several interventions currently used in clinical practice may indirectly influence the MuMi layer by putatively modulating mucus biology, microbial ecology, or inflammatory tone. This observation could support the MuMi layer as an emerging, albeit still indirect, therapeutic target.

## 7. Perspectives and Future Directions

**Figure 2:**
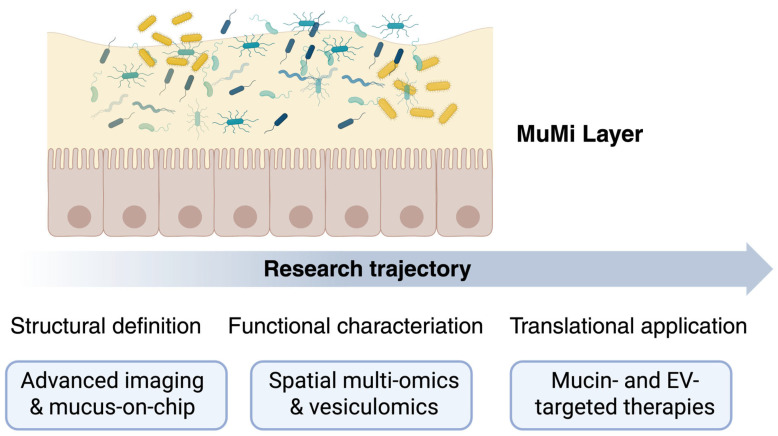
The MuMi layer as a dynamic barrier and signalling hub: from structure to clinical translation. The upper panel illustrates the core components of the gastroenteric MuMi layer: a mucus matrix (light yellow) that embeds the mucus-associated microbiota (coloured elements) above the epithelial surface (orange cells). This arrangement highlights the integrated nature of mucins, microbial residents, and host factors in maintaining mucosal homeostasis. The lower trajectory depicts the progressive stages of investigation: structural definition (advanced imaging and mucus-on-chip technologies enabling high-resolution analysis of mucus architecture and microbial localisation); functional characterisation (spatial multi-omics and vesiculomics revealing metabolic and signalling networks within the MuMi layer); and translational application (development of mucin- and EV-targeted therapeutic strategies for restoring MuMi integrity in disease). Overall, the figure emphasises the key role of multidisciplinary integration—from biophysics to clinical research—in decoding the MuMi layer as a unified eco-physiological system and a novel target for mucosal health interventions.

Despite significant advances in the characterisation of mucins, mucosa-associated microbiota and extracellular vesicles, the MuMi layer remains a largely unexplored frontier of mucosal biology. Future research must address the mechanistic, methodological and translational gaps that currently limit our understanding of this complex ecosystem. Figure 2 summarises our view of the conceptual evolution of MuMi-layer research, from structural definition to translational application.

From a mechanistic perspective, high-resolution approaches are needed to delineate how the structural organisation of mucus, its glycan landscape and the microbial consortia embedded within it co-evolve under physiological and pathological conditions. Recent evidence suggests that mucus glycans are not static barriers but dynamic regulators of microbial ecology and immune dialogue [19]. Integrating glycomics, microbiomics and vesiculomics at spatial resolution will be essential to map the functional architecture of the MuMi layer across regions of the gastroenteric tract. Furthermore, an additional level of complexity likely arises from age-dependent variations, as the composition and function of the MuMi layer may differ between children, adults, and the elderly. Understanding these potential differences represents an important, yet largely unexplored, avenue for future research.

Technological innovations such as mucus-on-chip and organoid-based mucus models are likely to play a pivotal role in dissecting MuMi-layer dynamics in controlled environments [64]. These systems allow real-time observation of mucin secretion, microbial adherence, vesicle trafficking and barrier permeability under defined chemical or mechanical stimuli. Likewise, advanced imaging and spatial transcriptomics [65] can help resolve microgradients in pH, oxygen, and nutrients that shape MuMi-associated microbial niches. The combination of these approaches with longitudinal sampling in human cohorts will enable causal inference between MuMi-layer alterations and disease states, rather than merely correlating them.

Translationally, the MuMi layer offers promising opportunities for biomarker discovery and therapeutic intervention. Altered mucin glycosylation, shifts in mucus-associated microbial taxa and changes in vesicle cargo composition may serve as early indicators of mucosal inflammation or neoplasia [66]. Strategies to restore MuMi-layer integrity may include targeted prebiotics, mucus-resident probiotics, dietary glycan supplementation, and engineered vesicles that deliver protective or immunomodulatory molecules, but we still lack preclinical models to investigate all these approaches. Establishing reproducible methods for mucus sampling, quantitative analysis of mucins, and profiling of mucus-associated microbiota and vesicles will be a prerequisite for translational progress.

Conceptually, the MuMi layer should be regarded as an integrated and dynamic barrier system whose perturbation may contribute to a broad spectrum of disorders, including inflammatory bowel disease, metabolic syndrome, infection susceptibility and colorectal carcinogenesis. Its study therefore requires a multidimensional framework encompassing microbiology, glycobiology, immunology and systems medicine. In the coming years, the field will need to prioritise the creation of reference atlases of MuMi-layer composition across the gastroenteric tract, the harmonisation of nomenclature and methodologies, and the design of interventional trials that incorporate MuMi-related biomarkers as readouts. Future studies should also consider age-specific variations in MuMi-layer composition and function, which may have implications for personalised strategies in mucosal health and disease prevention. Only through such coordinated efforts will it be possible to translate the MuMi concept into clinical applications and to extend it beyond gastroenterology, providing a unifying paradigm for mucosal health across organ systems.

## 8. Conclusions

Understanding the gastrointestinal wall as a structurally and functionally integrated system is crucial for grasping how health is maintained and how disease emerges. The concept of the MuMi layer we propose arises from recognising that the mucus barrier and its associated microbiota operate not as two distinct entities, but as a continuous, homeostatic interface between the host and the luminal environment. Within this layer, mucins, commensal microorganisms, secreted vesicles, and immune mediators interact dynamically, shaping a self-regulating ecosystem that sustains epithelial integrity, metabolic balance, and immune tolerance.

When the homeostasis of this eco-physiological unit is perturbed—by inflammation, infection, diet, or metabolic stress—the consequences extend beyond the mucosa itself. Alterations in the MuMi layer architecture can propagate along the gut–systemic axes, influencing not only gastrointestinal diseases but also extra-intestinal disorders linked to chronic low-grade inflammation and barrier dysfunction. Thus, the structural and functional understanding of this layer becomes essential not only for deciphering disease mechanisms but also for designing physiologically relevant experimental and therapeutic models.

Our group developed the MuMi layer concept with a deep conviction that anatomical knowledge and functional insight are inseparable. One cannot fully understand physiology—or pathophysiology—without a precise appreciation of tissue organisation in living systems. Ignoring the presence and role of such an active, interactive barrier may obscure key steps in the pathogenesis of alimentary and systemic disorders and limit the translational relevance of experimental models that fail to reproduce its structural complexity.

As Hippocrates famously stated, *“We are what we eat.”* Today, this ancient wisdom can be revisited in light of modern biology: *we are what we become through eating—thanks, in large part, to the homeostatic filter of the mucosal-microbiotic layer.*

## Data Availability

Not applicable.
